# Controlling hazardous chemicals in microreactors: Synthesis with iodine azide

**DOI:** 10.3762/bjoc.5.30

**Published:** 2009-06-12

**Authors:** Johan C Brandt, Thomas Wirth

**Affiliations:** 1 Cardiff University, School of Chemistry, Park Place, Cardiff CF10 3AT, UK.

**Keywords:** azide, flow chemistry, hazardous reagents, microreactor, rearrangement

## Abstract

Aromatic aldehydes have been converted into the corresponding carbamoyl azides using iodine azide. These reactions have been performed safely under continuous flow reaction conditions in microreactors.

## Introduction

Microstructured devices have already found their way into organic synthesis, because they offer various advantages over traditional large-scale chemistry performed in flasks or vessels [1–2]. The high surface-to-volume ratio as their main characteristic can result in rapid mixing of compounds, uniform reaction conditions due to precise temperature control as well as rate enhancements because of short diffusion distances. In addition, the synthesis and use of potentially hazardous compounds is another advantage of microreactors as only very small amounts of compounds/reagents are handled. Large inventories of dangerous reagents and intermediates are not necessary.

Azides are among the most versatile reagents in modern organic chemistry however they are not often used to their full potential due to safety concerns. Especially azides with low molecular weights are difficult to handle because of their high disposition to detonate [3–5]. However, azides are extremely useful moieties in organic synthesis as they can be transformed easily into a large variety of other functional groups [5].

Iodine azide is a very hazardous but valuable reagent that is easier and much more safely handled under microreactor conditions. Iodine azide is a solid compound, highly explosive and toxic. It is known to add stereospecifically to carbon–carbon double bonds with high regioselectivity following an ionic mechanism. This azido-iodination reaction is by far the most common synthetic application of iodine azide [6–14]. Due to the weakness of the iodine–nitrogen bond iodine azide also reacts in a radical manner upon heating, where this weak bond can be homolytically cleaved and introduce azide moieties efficiently into molecules with weak carbon–hydrogen bonds such as benzyl ethers [15–16] or aldehydes [17–18]. Recently some other research groups have investigated the use of azides in chemistries performed in microreactors [19–22].

## Results and Discussion

**Scheme 1:**
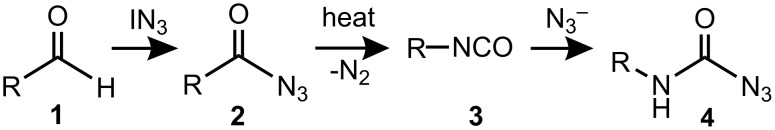
Azide addition to aldehydes and formation of carbamoyl azides.

The radical addition of azide to aldehydes **1** initially forms acyl azides **2** which directly rearrange in a Curtius rearrangement to the corresponding isocyanates **3**. Isocyanates are very reactive themselves and offer a wide range of possible conversions. An excess of azide leads to the formation of stable carbamoyl azides **4** as shown in Scheme 1.

**Figure 1:**
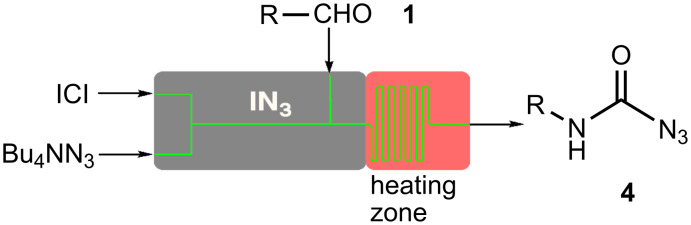
Microreactor setup for the *in situ* generation and use of iodine azide (IN_3_).

As sodium azide is poorly soluble in organic solvents, we decided to use tetrabutylammonium azide for the *in situ* generation of iodine azide. The reaction with iodine monochloride is rapid and after several minutes (depending on the flow rate) the aldehyde **1** was added to the reagent in the microreactor as shown in Figure 1.

All reagents and the aldehyde **1** were used as solutions in acetonitrile. After mixing the aldehyde with the iodine azide reagent the solution is passed through a capillary (volume: 196 µL) in a heating zone to allow the Curtius rearrangement to occur. Flow rates of 20 µL/min or higher led to very low or no conversion. At slower flow rates the yields were enhanced but seemed to stop growing at one point. Optimal yields were constant within a range of 15 to 1.5 µl/min corresponding to residence times in the heating zone of 13–130 minutes. For optimization experiments benzaldehyde **1a** was used. Slower flow rates are technically possible but not very attractive from a synthetic point of view. The reaction would work best at 80 °C, just below the boiling point of acetonitrile. Lower temperatures gave lower conversions. A reaction at 65 °C produced the product **4a** in only 25% yield, whereas a reaction at room temperature did not yield any product. An exchange of the solvent for propionitrile allowed a temperature increase to 110 °C, but conversions were found to be similar to reactions using acetonitrile. In the original publication the solvent had already been optimized and acetonitrile seemed to be the best solvent for this reaction [17].

When using non-distilled *p*-bromobenzaldehyde **1c** the reaction stopped with the formation of the acyl azide **5** contrary to the corresponding chloride derivative (Table 1). However when we modified the protocol to only using freshly distilled aldehydes, we also observed reaction and rearrangement of **1c** via **5** to **4c** and increased yields for the other reactions.

**Table 1:** Products and yields in the addition of iodine azide to aldehydes performed in a tubing microreactor at a flow rate of 7.5 µL/min (26 min residence time) at 80 °C.

Entry	Aldehyde **1**	Product	Yield [%]

1	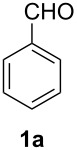	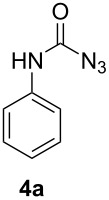	44
2	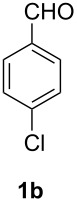	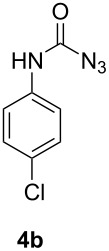	32
3	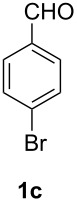 not distilled	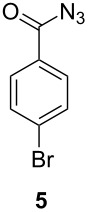	24^a^
4	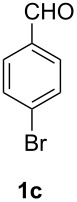 distilled	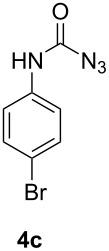	27
5	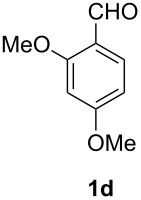	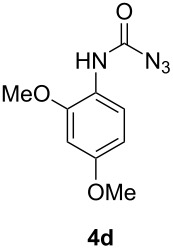	21

^a^No carbamoyl azide is obtained when crude (non-distilled) aldehyde **1c** is used.

We could show that the quality of the tetrabutylammonium azide is important for the success of the reaction. Its preparation from tetrabutylammonium hydroxide and sodium azide in water and dichloromethane did not lead to dry, crystalline material [23]. Unfortunately also the procedure using tetrabutylammonium chloride and sodium azide produced a product highly contaminated with DMF, which was not removable without decomposition of the product [24–25]. Highest yields were obtained with commercially available tetrabutylammonium azide (Aldrich), although great care has to be taken as this compound is very hygroscopic. Calculations indicate that the synthesis of iodine azide from trimethylsilyl azide and iodine monochloride is slightly endothermic (ΔH ≈ +15 kJ/mol) [26–29]. Geometry optimisations and frequency analyses on Me_3_SiN_3_, ICl, IN_3_ and Me_3_SiCl were performed using Gaussian 98 at the B3LYP/6-311+G(d,p) level using the LANL2DZ basis set for iodine augmented with one p function and one d function. NMR experiments showed a ratio of about 1:3 of trimethylsilyl azide and trimethylsilyl chloride upon reaction with iodine monochloride. This mixture was not efficient in the reaction with aldehydes, only an unidentifiable mixture of products was obtained.

**Scheme 2:**
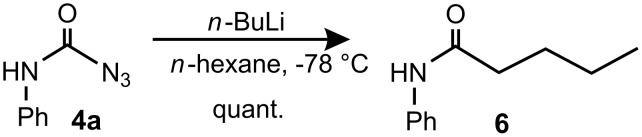
Reaction of carbamoyl azide **4a** with *n*-butyllithium.

The reactivity of carbamoyl azides is analogous to isocyanates, compound **4a** reacted with *n*-butyllithium to give amide **6** in quantitative yields as shown in Scheme 2.

Another way to form a stable iodine–azide bonds are hypervalent iodine compounds [30–31]. This source of reagent has indeed been used for radical azidonations in flask reactions [18] and resulted in an interesting approach using solid support [32], but these compounds are usually unsuitable for applications in microreactors because of their poor solubility or they would require a different microreactor set-up that would allow solid supported reagents [33].

For better comparison with literature procedures the reaction was then also investigated as a batch process. The reaction with commercial tetrabutylammonium azide and non-distilled aldehyde precursors would stop after the formation of the acyl azide with *p*-chlorobenzaldehyde and with *p*-bromobenzaldehyde. Rearrangement took place when sodium azide was used, but lower yields (34% yield of **4b**) were observed than those given in literature (53–97%) [17]. Maybe the sodium ion acts like a Lewis acid and accelerates the rearrangement. It is known that Lewis acid catalysts such as zinc triflate can accelerate Curtius rearrangements and the sodium cation might serve a similar role [34]. We obtained 67% yield of **4b** when zinc triflate was used as catalyst in a flask reaction. Even the reaction of non-distilled *p*-bromobenzaldehyde **1c** under these acidic conditions showed traces of the corresponding carbamoyl azide according to ^1^H NMR. But electron rich substrates such as 2,4-dimethoxybenzaldehyde **1d** showed decomposition and unidentifyable product mixtures with this catalyst. Generally, the batch reactions all proceeded less cleanly compared to the microreactor processes.

## Conclusion

After initially promising results we were challenged with a complex optimization task, that included many more parameters than usual in organic synthesis, due to the technical implications of our approach. There are phenomena in this reaction that prevent complete conversion to complete by a long way, probably azide-consuming side reactions or lack of activity of the substrate, lack of acid catalysis, or a combination of these reasons. Additionally, the substitution pattern of the substrates led to an unexpected outcome of the reaction.

## Experimental

### Synthesis of tetrabutylammonium azide:

**Procedure I **[23]: Sodium azide (200 mmol, 13 g) was dissolved in water (30 mL). Then tetrabutylammonium hydroxide (100 mmol, 26 g) was added and the mixture was stirred at room temperature for 90 min. Dichloromethane (50 mL) was added and stirring was continued for 10 min. The organic layer was separated and dried over magnesium sulfate. Finally the solvent was removed under reduced pressure and the product was dried for 24 h under vacuum.

**Procedure II **[24–25]: Sodium azide (79 mmol, 5.13 g) and tetrabutylammonium chloride (85 mmol, 22 g) were mixed in DMF (35 mL) and stirred overnight under argon at 50 °C. Dry THF (70 mL) was added and stirring continued for 15 min. The reaction mixture was filtered and the solvent in the filtrate was removed under reduced pressure. The product was heated under vacuum for 2 h to remove some DMF.

Yields for these reactions cannot be given as the solvents could not be removed completely from the product. IR (Nujol, cm^−1^): ν = 3418, 2145 (azide), 1643.

#### General procedure for the synthesis of carbamoyl azides **4**:

A twin syringe pump (KDS-200-CE) was charged with two 5 ml Hamilton syringes, one containing tetrabutylammonium azide (3 mmol, 853 mg) in dry acetonitrile (3.5 mL) and the other iodine monochloride (2 mmol, 102 µL) in dry acetonitrile (4.8 mL). The outlets were connected by standard HPLC tubing to a T-junction, after 30 cm tubing a second T-junction was connected to the third syringe containing the substrate aldehyde (1 mmol) in dry acetonitrile (2.4 mL). All HPLC equipment was purchased from CHM GmbH, Fridolfing, Germany. The HPLC tubing used as flow reactor was made of PTFE with the measurements 1/16 × Ø 0.25 mm. The syringes containing the reagents were connected to the flow system with PEEK adapters (1/4- 28f – Luer, female) and 1/4 PEEK fittings. The T-mixers used were PEEK (1/4-28 with 1/16 fittings and Ø 0.5 mm) for low pressure applications. The third syringe was loaded on a separate syringe pump. The flow rate was adjusted to an overall flow rate of 7.5 µL/min. The source of heating for the reaction was a PEG 600 oil bath. After passing the heating zone (80 °C) behind the second T-junction (4 m) the reaction was terminated at the outlet with 5 mol% aq thiosulfate solution. For work-up, the mixture was extracted with dichloromethane (3 × 10 mL). The combined organic phases were separated, dried over magnesium sulfate and the solvent removed under reduced pressure.

#### Phenylcarbamoyl azide (**4a**) 

The crude product was purified with a silica gel column using hexane/CH_2_Cl_2_ 15:1 as eluent. Yield: 71 mg (44%), colourless solid, mp: 106–107 °C (lit: 107.1 °C) [18].

^1^H NMR (500 MHz, CDCl_3_): δ 7.44 (d, ^3^*J*_HH_ = 7.9 Hz, 1H, H-2), 7.33 (t, ^3^*J*_HH_ = 7.9 Hz, 2H, H-1/H-3), 7.13 (t, ^3^*J*_HH_ = 7.4 Hz, 2H, H-4/H-6), 6.97 (s, 1H, N*H*) ppm; ^13^C NMR (125 MHz, CDCl_3_): δ 154.0 (C-8), 136.8 (C-5), 129.2 (C-1/C-3), 124.6 (C-2), 119.2 (C-4/C-6) ppm; EI-MS *m/z* (%): 162 ([M]^+^, 7), 119 (100), 93 (64), 91 (56), 64 (32).

#### 4-Chlorophenylcarbamoyl azide (**4b**)

The crude product was purified with a silica gel column using hexane/CH_2_Cl_2_ 1:1 as eluent. Yield: 63 mg (32%), colourless solid, mp: 103–105 °C.

^1^H NMR (500 MHz, CDCl_3_): δ 7.39 (d, ^3^*J*_HH_ = 8.8 Hz, 2H, H-4/H-6), 7.29 (d, ^3^*J*_HH_ = 8.8 Hz, 6.86 (s, 1H, N*H*) ppm; ^13^C NMR (125 MHz, CDCl_3_): δ 154.0 (C-9), 135.4 (C-5), 129.8 (C-2), 129.2 (C-1/C-3), 120.4 (C-4/C-6) ppm; EI-MS *m/z* (%): 198 ([M]^+^, 2), 196 ([M]^+^, 4), 187 (2), 185 (5), 155 (12), 153 (100), 127 (23), 125 (21); HRMS (EI): calc. for C_7_H_5_^35^ClN_4_O: 196.0146, found: 196.0145.

#### 4-Bromophenylcarbamoyl azide (**4c**)

The crude product was purified with a silica gel column using hexane/CH_2_Cl_2_ 1:1 as eluent. Yield: 65 mg (27%), colourless crystals, mp: 77–78 °C.

^1^H NMR (500 MHz, CDCl_3_): δ 7.45 (d, ^3^*J*_HH_ = 8.7 Hz, 2H, H-3/H-5), 7.34 (d, ^3^*J*_HH_ = 8.7 Hz, 2H, H-2/H-6) 6.84 (s, 1H, N*H*) ppm; ^13^C NMR (125 MHz, CDCl_3_): δ 154.1 (C-7), 136.0 (C-4), 132.2 (C-1), 120.9 (C-2/C-6), 117.4 (C-3/C-5) ppm; EI-MS *m/z* (%): 242 ([M]^+^, 100), 240 ([M]^+^, 96), 231 (25), 229 (27), 214 (10), 212 (19). HRMS (EI): calc. for C_7_H_5_BrN_4_O: 239.9641, found: 239.9639.

#### 2,4-Dimethoxyphenylcarbamoyl azide (**4d**)

The crude product was purified with a silica gel column using hexane/CH_2_Cl_2_ 1:4 as eluent. Yield: 47 mg (21%), colourless solid, mp: 65–67 °C.

^1^H NMR (500 MHz, CDCl_3_): δ 8.02 (d, ^3^*J*_HH_ = 8.6 Hz, 1H, H-4), 7.22 (s, 1H, N*H*), 6.50–6.47 (m, 2H, H-1/H-3), 3.84 (s, 3H, H-13 or H-14), 3.80 (s, 3H, H-13 or H-14); ^13^C NMR (125 MHz, CDCl_3_): δ 156.7 (C-10), 153.4 (C-2), 149.3 (C-6), 119.9 (C-4), 109.9 (C-3), 103.9 (C-5), 98.7 (C-1), 55.7 (C-13 or C-14), 55.5 (C-13 or C-14) ppm; EI-MS *m/z* (%): 222 ([M]^+^, 10), 179 (100), 164 (31), 153 (18), 136 (52), 122 (10), 110 (11), 95 (12); HRMS (EI): calc. for C_9_H_10_N_4_O_3_: 222.0753, found: 222.0755; IR (neat): 1531, 1711, 2144, 3415 cm^−1^.

#### Azido(4-bromophenyl)methanone (**5**)[35]

The crude product was purified with a silica gel column using hexane/CH_2_Cl_2_ 1:1 as eluent. Yield: 54 mg (24%), light yellow solid.

^1^H NMR (500 MHz, CDCl_3_): δ 7.87 (d, ^3^*J*_HH_ = 8.8 Hz, 2H, H-4/H-6), 7.59 (d, ^3^*J*_HH_ = 8.8 Hz, 2H, H-1/H-3) ppm; ^13^C NMR (125 MHz, CDCl_3_): δ 171.7 (C-8), 132.0 (C-5), 130.8 (C-1/C-3), 129.7 (C-2), 129.5 (C-4/C-6) ppm.

#### *N*-Phenylpentanamide (**6**) 

Phenylcarbamoyl azide **4a** (0.12 mmol, 20 mg) was dissolved in dry THF (35 mL) and cooled to −78 °C under argon. Then *n*-BuLi (0.37 mmol, 0.15 mL, 2.5 M in hexane) was added carefully dropwise into the solution via a syringe. After the addition the cooling bath was removed and the flask was allowed to warm to r.t. The mixture was then diluted with AcOH/THF (20 mL/20 mL) and aq sodium carbonate (20 mL). Then ethylacetate (60 mL) was added, the organic layer separated and dried over magnesium sulfate. The crude product was purified with a silica gel column using hexane/CH_2_Cl_2_ 15:1 as eluent. Yield: 14 mg (quant.) colourless solid, mp: 60–61 °C (lit: 60–61.5 °C) [36].

^1^H NMR (500 MHz, CDCl_3_): δ 7.51 (d, ^3^*J*_HH_ = 7.9 Hz, 2H, H-4/H-6), 7.32 (t, ^3^*J*_HH_ = 7.9 Hz, 2H, H-1/H-3), 7.13 (s, 1H, N*H*), 7.10 (t, ^3^*J*_HH_ = 7.4 Hz, 1H, H-2), 2.36 (t, ^3^*J*_HH_ = 7.6 Hz, 2H, H-10), 1.72 (qn, ^3^*J*_HH_ = 7.6 Hz, 2H, H-11), 1.41 (*sext*, ^3^*J*_HH_ = 7.5 Hz, 2H, H-12), 0.95 (t, ^3^*J*_HH_ = 7.4 Hz, 3H, H-13) ppm; ^13^C NMR (125 MHz, CDCl_3_): δ 171.3 (C-8), 137.9 (C-5), 129.0 (C1/C-3), 124.2 (C-2), 119.7 (C-4/C-6), 37.6 (C-10), 27.7 (C-11), 22.4 (C-12), 13.8 (C-13) ppm.
